# Letter from A. G. Smythe, M.D., of Baldwyn, Miss.

**Published:** 1874-05

**Authors:** A. G. Smythe

**Affiliations:** Baldwyn, Miss.


### CORRESPONDENCE.

BALDWYN, MISS., April 20, 1874.

*Editors Southern Medical Record:*

In the January number of THE SOUTHERN MEDICAL RECORD, page 46, there is a short article, "*Risks Attending the Use of Bromide of Potassium,*" with two cases reported to sustain the assertion. I am well satisfied that there are "*risks*" in the use of all active or patent remedies, but the risks in the use of a remedial agent ought not to deter a prudent use or trial of such remedies I do not intend to enter into any controversy upon this or any other subject. But wish to offer as a counterpoise a little experince which I have had in the use of the bromide of potassium. From several cases which I have used the article in question, freely, with similar results, will select a case.

Virgil G——, a stout lad, of about fourteen years, was attacked with manifest symptoms of epilepsy in the latter part of the year 1872. The symptoms increased rapidly up to the first of April, 1873. At which time he was having from one to three paroxysms every day. At this time he was presented at my office, for examination and treatment. Finding his general health good, I commenced the use of the pot. brom. in doses of twenty grains each, three times a day. He had but one paroxysm (some weeks after) from the time he commenced taking the remedy, his general health already good, continued to improve. He was anxious to labor but was not permitted to do so, his friends fearing that the exertion would cause a recurrence of the paroxysms. The bromide was taken daily, from April to the close of the year, (nine months) with the most happy effect, two pounds and four ounces having been used. He is now and has been engaged at common labor, from the first of January last, up to this (20th April) time, with no symptom of a return of the epilepsy, in good general health and fine spirits, with no treatment in three and a half months.

I will avail myself of this opportunity to say, that at one time I was not satisfied with the action of the bromide of potassium, not for its active results, but for what I conceived to be its negative results, from the representations made by what I conceived to be high authority I was led to expect too much and almost abandoned the use of it in disgust, and I might say in mortification and disappointment, but was induced to try it again. I am now convinced that I did not give it a fair trial, and without an attempt to consume time and space, or any theory, feel assured that it is one of the safest tranquilizers that we possess.

A. G. SMYTHE, M.D.

